# Targeting Tumor Acidosis and Regulatory T Cells Unmasks Anti-Metastatic Potential of Local Tumor Ablation in Triple-Negative Breast Cancer

**DOI:** 10.3390/ijms23158479

**Published:** 2022-07-30

**Authors:** Corrine A. Nief, Alana Gonzales, Erika Chelales, Júlia Sroda Agudogo, Brian T. Crouch, Smita K. Nair, Nirmala Ramanujam

**Affiliations:** 1 Department of Biomedical Engineering, Duke University, Durham, NC 27708, USA; alana.gonzales@duke.edu (A.G.); erika.chelales@duke.edu (E.C.); jagudogo@bidmc.harvard.edu (J.S.A.); brian.crouch@duke.edu (B.T.C.); nimmi@duke.edu (N.R.); 2 Stanford School of Medicine, Stanford University, Stanford, CA 94205, USA; 3 Obstetrics and Gynecology, Beth Israel Deaconess Medical Center, Boston, MA 02215, USA; 4 Obstetrics, Gynecology and Reproductive Biology, Harvard Medical School, Boston, MA 02115, USA; 5 Department of Surgery, Duke University School of Medicine, Durham, NC 27708, USA; smita.nair@duke.edu; 6 Department of Pathology, Duke University School of Medicine, Durham, NC 27708, USA; 7 Department of Neurosurgery, Duke University School of Medicine, Durham, NC 27708, USA; 8 Department of Pharmacology and Cancer Biology, Duke University, Durham, NC 27708, USA; 9 Duke Global Health Institute, Duke University, Durham, NC 27708, USA

**Keywords:** ablation, tumor microenvironment, immunomodulation, breast cancer, low-resource

## Abstract

Triple-negative breast cancer (TNBC) is an immunologically heterogenous disease that lacks clinically actionable targets and is more likely to progress to metastatic disease than other types of breast cancer. Tumor ablation has been used to increase response rates to checkpoint inhibitors, which remain low for TNBC patients. We hypothesized that tumor ablation could produce an anti-tumor response without using checkpoint inhibitors if immunosuppression (i.e., Tregs, tumor acidosis) was subdued. Tumors were primed with sodium bicarbonate (200 mM p.o.) to reduce tumor acidosis and low-dose cyclophosphamide (100–200 mg/kg i.p.) to deplete regulatory T cells, as has been shown independently in previous studies. A novel injectable ablative was then used to necrose the tumor, release tumor antigens, and initiate an immune event that could create an abscopal effect. This combination of bicarbonate, cyclophosphamide, and ablation, called “BiCyclA”, was tested in three syngeneic models of TNBC: E0771 (C57BL/6), 67NR (BALB/c), and 4T1-Luc (BALB/c). In E0771 and 67NR, BiCyclA therapy significantly reduced tumor growth and cured 5/7 and 6/10 mice 50 days after treatment respectively. In the metastatic 4T1-Luc tumors, for which surgery and checkpoint inhibitors fail, BiCyclA cured 5/10 mice of primary tumors and lung metastases. Notably, CD4+ and CD8+ T cells were found to be crucial for the anti-metastatic response, and cured mice were able to resist tumor rechallenge, suggesting production of immune memory. Reduction of tumor acidity and regulatory T cells with ablation is a simple yet effective therapy for local and systemic tumor control with broad applicability as it is not limited by expensive supplies.

## 1. Introduction

As a tumor’s metabolic demand outgrows its local blood supply, a hypoxic and acidic microenvironment is created around the tumor as a result of pro-glycolytic metabolic reprogramming and subsequent lactic acid accumulation [1,2]. This hostile tumor microenvironment suppresses anti-tumor immune cells [3] and allows pro-tumor immune cells such as regulatory T cells (Tregs) and tumor-associated macrophages (TAMs) to thrive [1,4]; thus, the tumor microenvironment is a promising target for anti-cancer therapies. Tumor acidosis promotes immune evasion and disease progression, and has recently been found to upregulate checkpoint signaling that dampens the effect of checkpoint inhibitor therapy [5]. Only a small subset of patients respond to checkpoint inhibitors, and tumor acidosis has been found to predict response rates to immunotherapies [3]. One cancer type that has had subpar response rates to immunotherapies is triple-negative breast cancer (TNBC). TNBC is an aggressive breast cancer type without curative therapies that disproportionately affects black and African women [6]. In high-income countries, PD-L1-positive TNBC patients can benefit from access to immune checkpoint inhibitors; however, only a small portion of TNBC patients have PD-L1-positive tumors, and notably these therapies are prohibitively expensive [7] for widespread utilization globally.

Even more troubling is the fact that an estimated 9 out of 10 people (estimated to be 5 billion) in low-income countries lack access to basic surgery [8], the most common frontline treatment for breast cancer. We are faced with a public health crisis: low-resource settings are expected to bear the brunt of the global cancer burden [9], yet they only have 5% of the available resources to combat this devastating group of diseases [10]. To make matters even worse, breast cancer is often diagnosed at advanced stages in these countries [11], owing to limited access to screening and diagnosis. As a result, more than two-thirds of the 10 million annual cancer deaths occur in countries with low income [12], and this number is expected to double by 2040 [9]. There is a dire need for early screening programs and non-surgical approaches for breast cancer treatment in resource-limited areas. One potential method for non-surgical tumor elimination is ablation.

Tumor ablation is the destruction of a tumor through temporary extreme conditions (i.e., heat, cold, chemicals) and is an attractive low-cost alternative for primary tumor control when surgery is impractical. Additionally, several types of ablations have been shown to elicit an anti-tumor immune response through the release of tumor-associated antigens (TAAs) following tumor necrosis [13,14]; thus, systemic control could be achieved by a local ablation with a robust induction of the immune response. Tumor ablation paired with checkpoint inhibitors is currently being studied in many active clinical trials with varied success; however, checkpoint inhibitors remain cost prohibitive. Chemical ablation through percutaneous ethanol injection (PEI) was amongst the first tumor ablation methods since it was pioneered for use in the liver 40 years ago [15]. The benefit of PEI is its simplicity —low-cost with few tools required, and it can be used near large vessels, unlike thermal ablation. The main shortcoming of PEI is incomplete ablation due to ethanol leakage [16,17], one of the primary reasons PEI has been replaced in high-income settings by energy-based methods such as radiofrequency ablation. In this report, we utilize a novel formulation in which ethanol is mixed with an ethanol-soluble, water-insoluble polymer called ethylcellulose. Ethylcellulose-ethanol, or ECE, undergoes a liquid-to-solid phase change in aqueous media, forming a fibrous gel upon injection into tissue [18,19]. ECE ablation has been shown to produce larger, more compact necrotic zones in several preclinical models that afforded longer overall survival compared to traditional PEI [20,21].

We investigated ECE ablation in combination with other low-cost agents that alter systemic tumor-induced immunosuppression to control disease progression. We devised a treatment strategy using oral sodium bicarbonate, low-dose cyclophosphamide, and ECE ablation (together termed “BiCyclA”). Cyclophosphamide (cyclo) is a chemotherapeutic agent that has been known for decades to selectively reduce regulatory T cell (Treg) populations in both patients and mice [22]. Previous studies pairing cyclo with local ablation in mice have demonstrated selective Tregs elimination and induction of systemic antitumor immunity [23,24]. Bicarbonate (bicarb) is a buffering agent that can reduce acidity within the tumor through oral administration [25]. Tumors that are very metabolically active produce massive amounts of lactic acid and shift their local tumor microenvironment to an acidic state where anti-tumor immune cells struggle to function. Bicarb administration can metabolically reprogram tumors and was shown to increase the intratumoral pH from 6.7 to 7.1 in 4T1 tumors [26] and B16 tumors [27]. Additionally, oral bicarb therapy has been shown to increase infiltration of CD8 T cells into the tumor microenvironment and decrease pH-dependent T-cell anergy [27,28].

Because TNBC consists of tumors with heterogenous immunophenotypes, we used three syngeneic murine TNBC cell lines with varying immunogenicity and metastatic potential across two mouse backgrounds to investigate the therapeutic efficacy of BiCyclA. We found that BiCyclA successfully controls both the primary tumor and reduces the metastatic burden across 67NR (locally invasive and immunogenic), and 4T1-Luc (metastatic, PD-L1 negative, and luciferase expressing) and achieves local tumor control in E0771 (non-metastatic) TNBCs. We show that the depletion of CD8+ T cells significantly reduces the anti-metastatic response to BiCyclA therapy and, conversely, BiCyclA induces resistance to tumor rechallenge. Finally, we demonstrate that BiCyclA can be integrated with surgery as a neoadjuvant therapy, further improving outcomes in the highly aggressive 4T1-Luc tumors. Taken together, our results provide proof-of-concept that BiCyclA can be used both alone and as an adjunct to surgery for the treatment of TNBCs.

## 2. Results

### 2.1. Combination of Bicarb, Cyclo, and ECE Ablation (BiCyclA) Reduced Tumor Growth and Increased Survival in 67NR and E0771 Tumors

**Figure 1:**
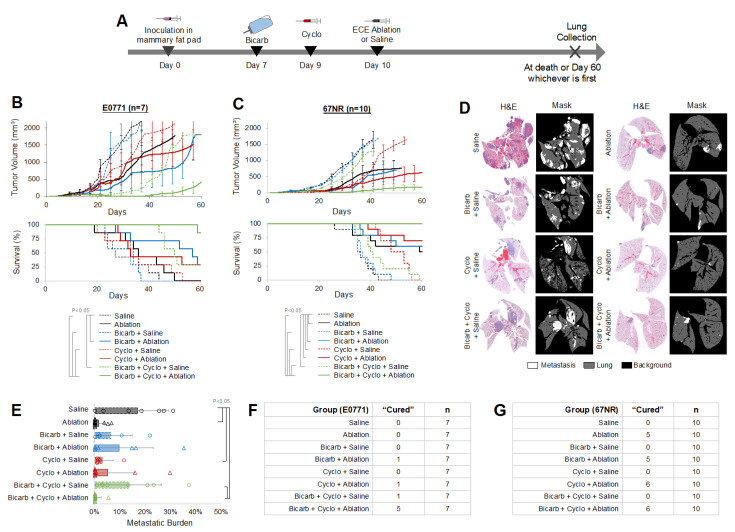
BiCyclA therapy was effective across different cell lines and mouse backgrounds. (**A**) Study timeline schematic for treatment of orthotopic 4T1-luc tumors. (**B**) Primary tumor volume and Kaplan–Meier survival curves for mice bearing E0771 tumors following treatment with BiCyclA (bicarb + cyclo + ablation) or controls. (**C**) Tumor growth and Kaplan–Meier survival curves for mice with 67NR tumors. (**D**) Representative images of whole-lung H&E and quantification mask for each treatment group in mice with 67NR tumors. (**E**) Metastatic burden quantified from the metastatic mask for each group. (**F**) Number of E0771 mice with no detectable tumor at the end of the study (*n* = 7). (**G**) Number of mice bearing 67NR tumors out of *n* = 10 that were without primary tumor or detectable metastases by the end of the study. Brackets indicate *p* < 0.05.

E0771 (C57BL/6 background) and 67NR (BALB/c background) tumors were treated using combinations of bicarb, cyclo, and either ECE ablation or saline (control). The combination of bicarb + cyclo + ablation is referred to as BiCyclA in the text. The timing of treatment is shown in Figure 1A. E0771 tumors are non-metastatic and are PD-L1 positive. 67NR tumors are “mildly” metastatic and PD-L1 positive [29]. Tumor growth and survival is seen in Figure 1B for E0771 tumors and Figure 1C for 67NR tumors. In both tumor lines, BiCyclA treatment resulted in the greatest reduction in primary tumor growth, and after BiCyclA treatment, most mice had no detectable primary tumor (7/10 for 67NR, 5/7 for E0771). Survival was limited by primary tumor burden in both tumor lines. Mice treated with BiCyclA lived longer than mice treated with any other therapy combination. At necropsy, whole lungs were collected for H&E staining, shown in Figure 1D and quantified in Figure 1E. No detectable micro-metastases were seen in the lungs of mice with E0771 tumors, so they were not analyzed further. Mice with 67NR tumors had the lowest average metastatic burden after BiCyclA, which was significantly better than saline controls. Of note, 67NR tumors are traditionally considered non-metastatic or micro-metastatic because their survival is limited by primary tumor and not metastatic disease; however, the lungs of mice with 67NR tumors are rarely analyzed microscopically. The decrease in primary tumor growth and fewer metastases conferred a significant survival advantage for both 67NR and E0771 mice. Mice with no primary tumor or detectable metastases on H&E were considered “cured”, and in E0771 tumors, BiCyclA therapy produced 5/7 cures (Figure 1F). Interestingly, in 67NR tumors, several treatment groups resulted in high numbers of cures including ablation (5/10), bicarb + ablation (5/10), cyclo + ablation (6/10), and BiCyclA (6/10), as seen in Figure 1G. These results suggest that “immunogenic” tumors such as 67NR and E0771 can be cured with combined BiCyclA therapy and there is some effect of each of the components.

### 2.2. BiCyclA Therapy Cured Metastatic 4T1-Luc Tumors

**Figure 2:**
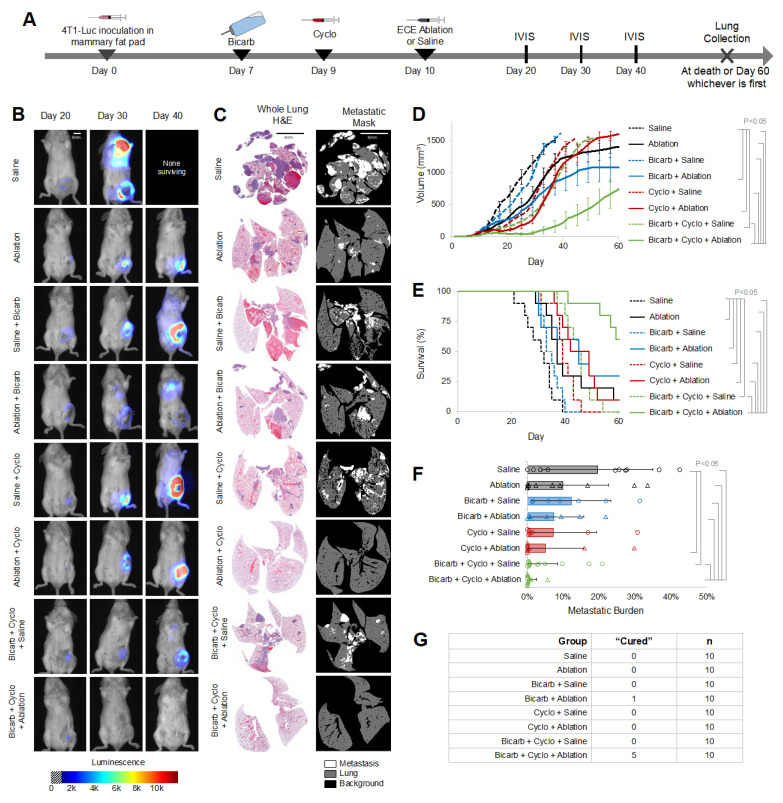
BiCyclA therapy reduced 4T1-luc tumor burden both locally and systemically. (**A**) Study timeline schematic for treatment of orthotopic 4T1-luc tumors. (**B**) Representative IVIS images from each treatment group with 4T1-luc tumors. (**C**) Representative whole-lung H&E and corresponding metastatic mask showing metastatic lesions in the lungs. (**D**) Average tumor volume over time. (**E**) Metastatic burden, or percent of lung area occupied by metastatic lesions, for each treatment group. Black bar represents average. (**F**) Survival rates for mice bearing 4T1-Luc tumors, *n* = 10. (**G**) Number of mice out of 10 per treatment group with no detectable metastases or primary tumor at day 60. Scale bar equals 5 mm in images.

While BiCyclA was able to cure a majority of 67NR and E0771 tumors, the effect of BiCyclA on highly metastatic tumors was unknown. We chose to assess BiCyclA and each of its components in 4T1-luc tumors, which are highly aggressive and are known to be resistant to common therapies (radiation, surgery, checkpoint inhibitor, etc.). Additionally, 4T1-Luc tumors express luciferase, which allows for longitudinal in vivo imaging of systemic of the disease. Mice were treated in accordance with the study timeline shown in Figure 2A. Control groups included the exhaustive combinations of bicarb, cyclo, and ECE ablation or saline as previously shown in Figure 1A. Representative IVIS images captured on days 20, 30, or 40 following tumor inoculation and treatment for each group are shown in Figure 2B. A representative image is not shown for saline control mice on day 40 because the only mouse surviving to day 40 in this group was found dead before imaging could be performed and gross metastases were seen on necropsy of this mouse. The IVIS images revealed significant local and systemic disease across all therapy combinations when ECE ablation was excluded. BiCyclA therapy performed better than the other combinations of treatments with approximately half of primary tumors regressing, and most mice avoided the development of metastatic disease. Figure 2C shows representative H&E sections and metastatic masks for lungs removed from treated 4T1-luc tumors at the time of euthanasia (either when a humane endpoint was reached or at day 60, whichever occurred first). The fewest number of metastases were observed in mice receiving BiCyclA therapy. Tumor volume, shown in Figure 2D, was significantly lower following BiCyclA therapy compared to all other groups. Similarly, the metastatic burden, defined as the percentage of the lung covered with metastases, was the lowest for BiCyclA therapy, as shown in Figure 2F. Figure 2G shows the number of cured mice in each group. Survival was typically limited by primary tumor growth for most treatment groups. Mice were considered cured if they had no primary tumor or evidence of metastatic disease on the basis of H&E analysis of the lungs at day 60. BiCyclA therapy resulted in 5/10 mice cured. The only other therapy combination to produce a cure was ECE ablation with bicarb, which cured 1/10 mice.

### 2.3. CD8+ Cells Contributed to the Anti-Metastatic Effects of BiCyclA Therapy

**Figure 3:**
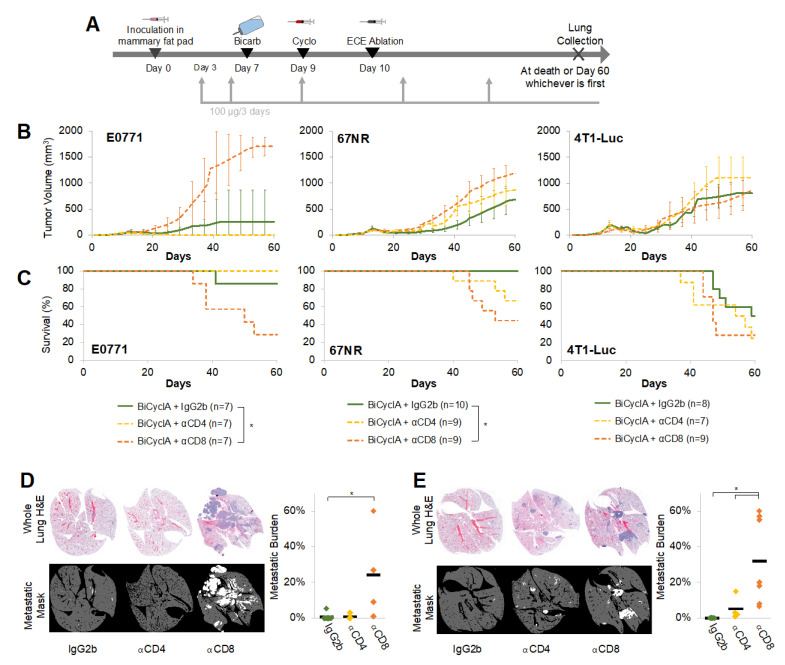
CD8+ cells contributed to the anti-metastatic effects of BiCyclA therapy. (**A**) Depletion study timeline. (**B**) Tumor volumes and (**C**) survival rates after bicarb + cyclo + ablation with or without depletion for mice with E0771, 67NR, and 4T1-Luc tumors. (**D**) Representative H&E, masks, and metastatic burden for mice bearing 67NR tumors. (**E**) Representative H&E, masks, and metastatic burden for mice bearing 4T1-Luc tumors. * *p* < 0.05; KS test (**B**) or Student’s *t*-test (**C**,**D**).

T-cell immunity has been shown to have a significant role in the anti-tumor effect produced by other ablative methods [30,31]; therefore, we hypothesized that CD8+ T cells could be responsible for the anti-tumor effects observed after BiCyclA. CD8+ and CD4+ cells were depleted in mice bearing E0771, 67NR, and 4T1-Luc tumors before BiCyclA therapy, as shown by the gray arrows in Figure 3A. Control mice received an IgG2b isotype control antibody. Starting on day 3, 100 μg of antibodies was injected intraperitoneally for each mouse. CD8+ cell depletion shortened survival compared to controls (Figure 3B–C) in E0771 and 67NR tumors; however, there was no significant difference in survival in the 4T1-Luc tumors, regardless of T-cell depletion. Figure 3D,E shows images of lung H&E following euthanasia either at a humane endpoint or 60 days post-treatment, whichever occurred first, as well as metastatic quantification in mice bearing 67NR and 4T1-Luc tumors, respectively. Mice with CD8+ cells depleted had a significantly higher metastatic burden than isotype control mice for both 67NR and 4T1-Luc tumors, suggesting that CD8+ T cells are important for distant tumor control following BiCyclA treatment.

### 2.4. Mice Cured with BiCyclA Therapy Resisted Tumor Rechallenge

**Figure 4:**
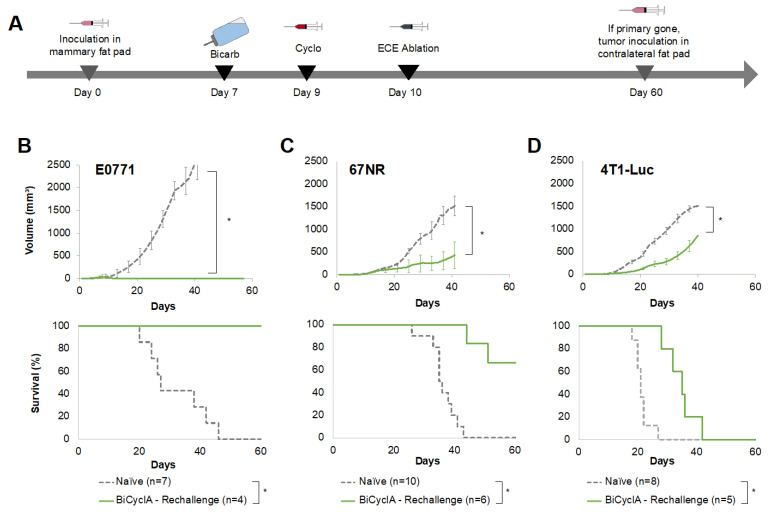
Mice cured with BiCyclA therapy resisted tumor rechallenge. (**A**) Study design for mice rechallenged with tumors following cure with BiCyclA (bicarb + cyclo + ablation). Tumor growth and survival in naïve mice and mice rechallenged with (**B**) E0771, (**C**) 67NR, and (**D**) 4T1-Luc tumors. * *p* < 0.05, KS test.

After finding that depletion of T cells hindered the anti-metastatic effect of BiCyclA therapy, we next investigated whether treatment with BiCyclA induced a lasting anti-tumor immunity through a tumor rechallenge experiment. Mice bearing E0771, 67NR, or 4T1-Luc tumors were treated with BiCyclA therapy and were followed for primary tumor growth and metastases. Mice with no evidence of disease at 60 days post-treatment were challenged in the contralateral mammary fat pad with the same tumor type, as shown in Figure 4A. Evidence of disease was defined as no palpable primary tumor, no luciferase signal seen with IVIS for 4T1-Luc tumors, and no sign of cachexia (body weight maintained from beginning of study). No additional interventions were provided following tumor inoculation on day 60. Tumor growth and survival for rechallenged mice compared to naïve tumor growth are shown in Figure 4B–D for E0771, 67NR, and 4T1-Luc mice, respectively. Tumor growth was significantly decreased, and survival was significantly increased in mice that had previously been cured using BiCyclA compared to naïve mice for all three tumor lines, suggesting a lasting anti-tumor response following BiCyclA therapy.

### 2.5. Tumor Ablation Was Crucial for Anti-Tumor and Anti-Metastatic Response in 4T1-Luc Tumors

We hypothesized that ECE ablation was initiating a systemic anti-metastatic response, but up until this point, we could not conclude whether ECE ablation simply destroyed the primary tumor and prevented the dissemination of metastases, or if micro-metastases were present at the time of treatment and were being actively destroyed. To test this hypothesis, we utilized 4T1-Luc tumors, which previous studies have shown to rapidly disseminate, with an orthotopic implantation of 7500 cells reliably producing micro- and macro-metastases in the lung, bone, and brain by 10 days after implant [32]. To determine whether the reduction of primary tumor eliminated the occurrence of metastases, we performed surgical excision on a group of controls.

**Figure 5:**
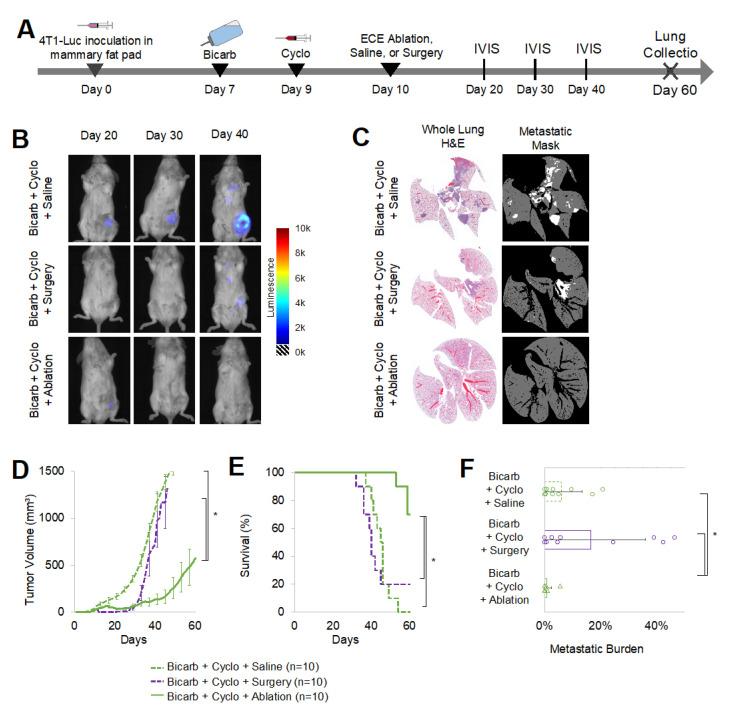
BiCyclA induced a more anti-metastatic response than surgery and could be integrated with surgical excision to control primary tumor growth. (**A**) Treatment timeline of ECE ablation, saline injection, and surgical excision in 4T1-Luc tumors. (**B**) Representative IVIS imaging of mice with 4T1-Luc tumors over time. (**C**) Representative whole-lung H&E and corresponding metastatic identification masks. (**D**) Average primary tumor growth for each treatment group. (**E**) Survival for each treatment group. (**F**) Metastatic burden for each group. * *p* < 0.05, KS test for (**D**,**E**), Student’s T-test for (**F**).

All mice received bicarb and cyclo, and on day 10, tumors were either surgically removed, given ECE ablation, or injected with saline, in accordance with the study design shown in Figure 5A. For the surgery group, tumor and draining lymph node were removed at day 10, and negative margins were confirmed with IVIS on the next day. 4T1-Luc tumors were visualized with IVIS imaging following treatment in all groups (Figure 5B), and lungs were collected at the time of death for H&E staining (Figure 5C). Tumor growth is shown in Figure 5D, and there were no detectable primary tumor lesions up to 10 days after surgery, while small lesions remained in the ablation group. Survival was significantly increased for the ablation group compared to those receiving surgical removal or saline injections (Figure 5E). Quantification of metastatic lesions at death revealed that BiCyclA therapy produced a significantly lower metastatic burden when compared to surgery with cyclo and bicarb (Figure 5F). Surgical removal of the tumor did not limit metastases, suggesting that ablation and not just primary tumor removal is required for the anti-metastatic effect of BiCyclA.

## 3. Discussion

Surgery, chemotherapy, and radiation therapy are the cornerstone treatments for TNBC. Unfortunately, despite best practices, many of these cancers ultimately recur as metastatic disease. Achieving lasting antitumor immunity following local therapy is an attractive goal for novel cancer therapeutics as it could eliminate both local and systemic disease. Targeting the immune checkpoint blockade with immune checkpoint inhibitors has shown promise in the treatment of a variety of cancer types [33]. Still, tumors that are immunologically “cold”, including PD-L1-negative TNBCs [34], show limited response to available immunotherapies. Approaches that immunologically engage the tumor thus converting a tumor from “cold” to “hot” could have significant clinical potential.

In this study, we tested the hypothesis that targeting tumor-induced immune suppression before local ablation could unlock an anti-tumor response in TNBCs without using cost-prohibitive therapies. While both cyclo and bicarb moderately increased rates of survival after ECE ablation, the combination of all three agents (bicarbonate, cyclophosphamide, and ECE ablation), which we refer to as BiCyclA, produced the most striking increase in survival across three murine models of TNBC, supporting our hypothesis. We found that local therapy, specifically ECE ablation and not surgical excision, was crucial for producing the systemic anti-tumor response to BiCyclA. Several other studies in both mice and humans have demonstrated that leaving tumor debris in situ can be beneficial for the development of a systemic anti-tumor immune response [13]. For example, a study using combination of an injectable hydrogel, thermal ablation, and low-dose cyclo demonstrated increased survival rates over each of the individual components in murine models [35].

Each tumor model used in this study had a different immunophenotype, and we found that the strength of the anti-tumor response after BiCyclA was, not surprisingly, model dependent. While BiCyclA was able to cure most mice with 4T1-Luc, 67NR, and E0771 tumors, only mice with E0771 tumors completely resisted tumor rechallenge. We hypothesize that the different levels of native immune cells in these tumor models is the source of therapeutic variability, which is supported by the variable results of the T-cell depletion study. For example, 4T1 tumors initiate expansion of myeloid-derived suppressor cells (MDSCs) in the host, which inhibits cytotoxic T-cell functionality, while both E0771 and 67NR tumors have very low amounts of MDSCs [36]. The T-cell depletion suggests that CD8+ and CD4+ cells play a complicated, and sometimes counterproductive, role in the anti-tumor response for different tumor models. Therefore, further research may be needed to characterize the mechanism for tumor-dependent responses to BiCyclA therapy.

Our results provide support for investigation of BiCyclA as an alternative or additive therapy in the TNBC treatment cascade across low- and high-resource settings. While BiCyclA is yet to be assessed in a clinical setting, each of the components of BiCyclA (cyclo, bicarb, ethanol, ethyl cellulose) are individually are already used clinically in many countries. Of note, translation of oral bicarb buffer therapy into humans has been controversial; however, a few small studies have demonstrated favorable results after intravenous bicarb delivery to tumors to improve response to trans-arterial chemoembolization (TACE) [37,38]. Our results corroborate their findings and demonstrate that ablation can be combined with pH-modulating therapies to improve the anti-tumor immune response.

BiCyclA can be contextualized for use in different clinical care paradigms as all the components are widely available in low- and high-resource settings. For example, in high-resource settings, BiCyclA could be used as a neoadjuvant treatment prior to surgery (Figure S1), as we demonstrated here, in combination with systemic therapies including checkpoint inhibitors (Figure S2) to prevent future recurrence. BiCyclA could be used as a stand-alone treatment when resources are scarce, or when tumors are small enough to be effectively treated with an ablative strategy. Given the ever-increasing rates of TNBCs in low-resource settings and the limited treatment options for these patients, the simplicity and broad efficacy of BiCyclA makes it an attractive therapy for further research and translation.

## 4. Methods

### 4.1. Tumor Cell Lines

Murine breast cell line 67NR [39] was provided by the Dr. Fred R. Miller laboratory (Karmanos Cancer Institute, Detroit, MI, USA) through Dr. Inna Serganova and Dr. Jason Koucher (Memorial Sloan Kettering Cancer Center, New York, NY, USA). 67NR tumors were verified with cytokeratin staining, CK8/18, by the Dr. Bob Cardiff laboratory (University of California Davis, Davis, CA, USA). 4T1-Luc and E0771 cell lines were provided by the Dr. Smita Nair lab (Duke University, Durham, NC, USA). All cell lines were routinely analyzed when frozen cultures were recovered for mycoplasma contamination using MycoAlert (Lonza) by the Immunology Virology Quality Assessment Center (IVQAC). Cells were not passaged more than 10 times after thawing from frozen. Both 67NR and 4T1-Luc cells were cultured in RPMI 1640 (VWR) with 10% FBS, 2 mM L-glutamine, 100 units/mL penicillin, and 100 mg/mL streptomycin. E0771 cells were cultured in RPMI 1640 (VWR) with 10% FBS, 2 mM L-glutamine, 10 mM HEPES, 100 units/mL penicillin, and 100 mg/mL streptomycin. Cells were cultured under standard conditions free of contamination at 37 °C, 5% CO_2_, and 95% humidity.

### 4.2. Murine Mammary Tumor Models

All animal work was approved by the Duke University Institutional Animal Care and Use Committee (IACUC) under protocol A160-18-07. The experimental unit was a single animal; the “n” reported in each figure is the number of mice used and thus the number of biological replicates. Mice were group housed in cages of 5 with standard chow and water ad libitum, unless otherwise specified. Mice were enriched with supplemental housing and bedding. 67NR and 4T1-Luc tumors were established in 5–6-week-old female BALB/c mice (Charles River Labs, Raleigh, NC, USA) through injection of 5 × 10^5^ cells in 100 μL of serum-free RPMI 1640 (without Matrigel) in the fourth mammary fat pad. A total of 100% of mice inoculated with 67NR and 4T1 cells developed tumors, and the sample size used for studies of these tumors was chosen to be 10 to provide more than sufficient statistical power. E0771 tumors were established in 5–6-week-old female C57BL/6 mice (Charles River Labs) through injection of 1 × 10^6^ cells in 100 μL of serum-free RPMI 1640 in the fourth mammary fat pad. Approximately 70% of mice inoculated with E0771 cells developed tumors, so a sample size of 7 was used for E0771 studies, which was still sufficiently powered. Mice were euthanized by CO_2_ inhalation and organ removal if tumors exceeded 15 mm in diameter or other humane endpoint (loss of at least 15% body weight, failure to groom, and self-mutilation).

### 4.3. Tumor Growth and Survival

For tumor growth and survival studies, treatment began as indicated by the study timeline in each figure. If there was no palpable tumor by the first day of treatment, the mouse was excluded from the study. Tumor volume (length × width^2^/2) was measured every 3 days following treatment by an observer that was blinded to the treatment group. Once the tumor exceeded 1500 mm^3^ (or any other human endpoint was met), the mouse was euthanized, and the final tumor volume recorded was used for tumor volume averages after death. Time to death or euthanasia was used to calculate Kaplan–Meier survival curves. Humane endpoints, such as loss of >15% body weight, paralysis, and cessation of grooming, were recorded as adverse events. If mice survived to 60 days without a tumor or any evidence of systemic disease, they were euthanized on day 60 to collect lungs for metastatic analysis. Evidence of disease was identified by body condition score below 3 and 15% drop in body weight as a sign of cancer cachexia. For mice bearing 4T1-luc tumors, IVIS imaging was also used to detect evidence of disease at 60 days.

### 4.4. 4T1-Luc Luminescent Imaging

4T1 tumors expressing luciferase (4T1-Luc) were used to allow for longitudinal monitoring of tumors and metastases. Mice were imaged using a whole-body, in vivo IVIS Lumina imaging system (Perkin Elmer: Waltham, MA, USA) in the supine position, 15 min after injection of 5 mg of D-luciferin (Sigma Aldrich: St. Louis, MO, USA) in 100 µL of sterile saline. Images were acquired with an exposure time of 5 s. Pixels with a luminescence reading of 600 or lower were considered background. All images were batch processed, and the treatment group was blinded. Area calculations were performed using standard image processing tools in MATLAB (MathWorks, Natick, MA, USA).

### 4.5. Bicarbonate-Cyclophosphamide-Primed Ablation (BiCyclA)

Mice bearing either E0771, 67NR, or 4T1-Luc tumors were randomized to receive one of eight treatment combinations: sodium bicarbonate (bicarb) with or without cyclophosphamide (cyclo), with or without ethylcellulose-ethanol (ECE) ablation. Cage location and order of treatment were randomized. Bicarb treatment (in which the only water available had 200 mM bicarbonate) could not be randomized on an inter-cage basis. Treatment began when tumors were palpable (≈50 µL in volume). Bicarb treatment was given by dissolving sodium bicarbonate to a concentration of 200 mM in mouse drinking water. Mice remained on 200 mM of sodium bicarbonate until day 60 or euthanasia at a humane endpoint, whichever occurred first. Cyclo (Sigma Aldrich, PHR1404, St. Louis, MO, USA) was given via intraperitoneal injection at a dose of 200 mg/kg for BALB/c mice and 100 mg/kg for C57BL/6 mice (C57BL/6 mice are more sensitive to cyclo). ECE ablations were performed via direct injection into the tumor. Specifically, mice were anesthetized with inhaled 1–3% *v*/*v* isoflurane in room air, mammary tumors were isolated by grasping with tweezers, and a 27G half inch needle was used to inject 6 mL/kg (100–150 µL) of the ablative solution by hand. Mice were monitored for adverse events for the hour after ablation. Anhydrous ethanol was obtained from Sigma Aldrich. USP-grade ethyl cellulose (Sigma Aldrich, SKU: 1265504) and USP-grade lidocaine (Sigma Aldrich, SKU: 1366002) were used for all ablations. Anhydrous ethanol was mixed with 10% w/w ethyl cellulose and 1% w/w lidocaine.

### 4.6. Mammary Tumor Excision Surgery

BiCyclA was both compared to and combined with tumor excision in 4T1-Luc tumor-bearing mice to compare BiCyclA to a standard therapy and demonstrate the feasibility of integrating BiCyclA with a standard therapy. Tumor excision was performed to mimic a lumpectomy surgery [40]. Briefly, an incision was made medial to the tumor, the tumor was isolated by blunt dissection, the feeding arteries were cauterized, the tumor was removed by visual inspection, the mammary lymph node was removed, and the skin was closed with silk sutures. Mice were given 5 mg/kg meloxicam SR for analgesia and were anesthetized with inhaled 2% isoflurane.

### 4.7. Lung Metastases Quantification

Lung metastases were quantified following BiCyclA treatment to investigate how BiCyclA affects metastasis formation. After euthanasia, lungs were perfused with 10% neutral buffered formalin and fixed for 24–48 h. Whole lungs were paraffin embedded and stained with hematoxylin and eosin by the Duke Substrate Core. Slides were scanned at 10x and stitched using a widefield microscope (Duke University, Light Microscopy Core Facility) on an Axio Imager Z2 Upright Microscope (ZEISS, Oberkochen, Germany). Images were compressed 95%, and a custom algorithm was used to automatically detect metastatic lesions and total lung volume, excluding blood and other tissues. Images were batch processed and thus blinded to treatment group. The algorithm’s accuracy was verified by a pathologist using 20 randomly selected raw images and masks. The “metastatic burden” was quantified as the area of metastatic lesions divided by total area of lung tissues.

### 4.8. T-Cell Depletion

CD4+ and CD8+ T cells were individually depleted to investigate the T-cell dependency of the BiCyclA treatment effect. Mice were injected intraperitoneally with 200 µL of sterile saline with 100 µg of antibody every 3 days, beginning 3 days after tumor inoculation. CD4+ T cells were depleted with anti-mouse CD4 (Clone GK1.5, BioXcell, Catalog # BE0003-1). CD8+ T cells were depleted with anti-mouse CD8α (Clone 2.43, BioXcell, Catalog # BE0061). As a control, one group of mice were given rat IgG2b isotype control, anti-keyhole limpet hemocyanin (Clone LTF-2, BioXcell, Catalog # BE0090).

### 4.9. Rechallenge

Mice with no evidence of disease at 60 days were re-challenged with 5 × 10^5^ tumor cells of the original cell line in the contralateral mammary fat pad. Mice were returned to water without sodium bicarbonate gradually starting on day 55, and by day 60, mice were on tap water. Tumor dimensions were recorded with calipers every 3 days until a humane endpoint was reached or 60 days post-implant, whichever occurred first.

### 4.10. Statistical Analysis

Statistical analyses were performed in MATLAB using built-in functions. Outliers were detected with a Grubbs test. No outliers were found to be excluded. Treatment groups were only revealed to the person administering treatment and were revealed for analysis after the data were collected. Of note, some treatments could not be completely blinded such as which mice were drinking bicarb, as they had a water bottle instead of the in-rack water, or when mice had surgery, as they had sutures on their body for a few days. Two-tailed ANOVA testing with unequal variance was performed on all groups between each other and controls with a 95% confidence interval. Post hoc multiple comparisons were performed using Tukey’s HSD test. Survival curves were quantified using Kaplan–Meier analysis, and a log-rank test was performed to determine the significance of a *p*-value less than 0.05 with a confidence level of 95%.

## 5. Conclusions

Here we demonstrate proof-of-concept for combining ethanol-based ablation, ECE, with low-dose cyclophosphamide and oral bicarbonate buffer therapy, referred to as BiCyclA. This combination targets tumor acidosis and Tregs to promote anti-tumor immunity after ablation. BiCyclA was found to be effective at reducing primary tumor volumes in 67NR and E0771 and at decreasing metastatic formation in the aggressive 4T1 model. This study suggests that bicarbonate buffering and low-dose cyclophosphamide can promote favorable outcomes after tumor ablation.

## Supplementary Materials

The following supporting information can be downloaded at https://www.mdpi.com/article/10.3390/ijms23158479/s1.
