# Supplementary material for: Targeting Tumor Acidosis and Regulatory T Cells Unmasks Anti-Metastatic Potential of Local Tumor Ablation in Triple-Negative Breast Cancer
Source: Int J Mol Sci. 2022 Jul 30;23(15):8479. doi: 10.3390/ijms23158479 (PMC9368760; doi:10.3390/ijms23158479)
Supplement: Supplementary file 1 [file ijms-23-08479-s001.zip › ijms-1837818-supplementary.pdf]

6. **Supplemental**

6.1 Use of BiCyclA as neoadjuvant therapy

We used 4T1-Luc cells to demonstrate that BiCyclA can be integrated with surgical excision of the primary tumor, to further slowdown primary tumor growth and improve survival since BiCyclA has a strong anti-metastatic response and the combination with surgery should improve the primary tumor response. This scenario is representative of a clinical scenario in which neoadjuvant therapy is performed prior to surgery. Traditionally, 4T1-Luc cells are resistant to treatment with surgery alone, showing primary tumor recurrence and metastasis formation; therefore, we compared BiCyclA and post-ablation surgery to surgery alone to determine if BiCyclA prior to surgery could further extend survival with 4T1-Luc tumors. The experiment methodology is shown in Figure S1A. Clear margins were confirmed using IVIS imaging after surgery. Control groups included treatment with ablation alone, surgery alone, bicarb+cyclo+surgery, and BiCyclA therapy. Figure S1B shows the primary tumor growth and overall survival curves, and Figure S1C shows the percentage of mice cured in each group. Mice receiving BiCyclA and post-ablation surgery lived significantly longer than mice given surgery alone, even though the surgery only group received surgery one week earlier than the BiCyclA + surgery group.

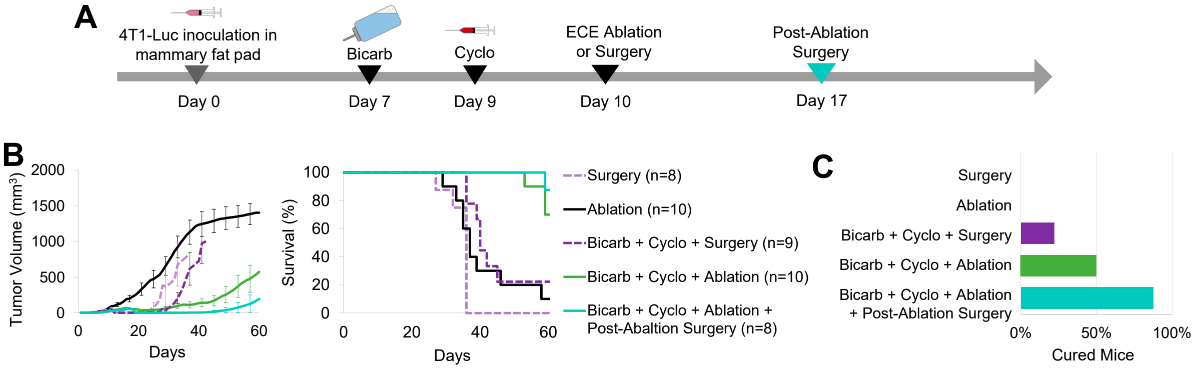

Figure S1: (**A**) Treatment timeline for Bicarb + Cyclo + Ablation + Surgery and Surgery alone. (**B**) Primary tumor growth and survival rates for mice given either Surgery alone or Bicarb + Cyclo + Ablation + Surgery. (**C**) Percent of mice cured by each treatment. *P<0.05 from Log-Rank test on Kaplan Meier Curves.

6.2 BiCyclA increases survival over CPI in 4T1-Luc tumors

While novel immunotherapies have been approved to treat some TNBCs, most TNBC patients have PD-L1 negative tumors which disqualifies them from benefiting from checkpoint inhibitors (CPI). Furthermore, CPIs are cost prohibitive for many patients. A regimen of anti-PD1 and anti-CTLA4 was given in mice bearing 4T1-Luc tumors and compared to BiCyclA as shown in the timeline in Figure S2A. 4T1-Luc bearing mice were given10 mg/kg anti–PD-1 (BioXcell) and anti–CTLA-4 (BioXcell) antibodies intraperitoneally on days 12, 15, and 17 after tumor implantation [41]. Tumor growth

and survival is shown in Figure S2B. 4T1-Luc tumors were resistant to treatment with CPI while BiCyclA resulted in a delay in tumor growth. Adding BiCyclA to CPI increased survival compared to CPI alone, however survival after BiCyclA + CPI was not significantly different than after BiCyclA alone.

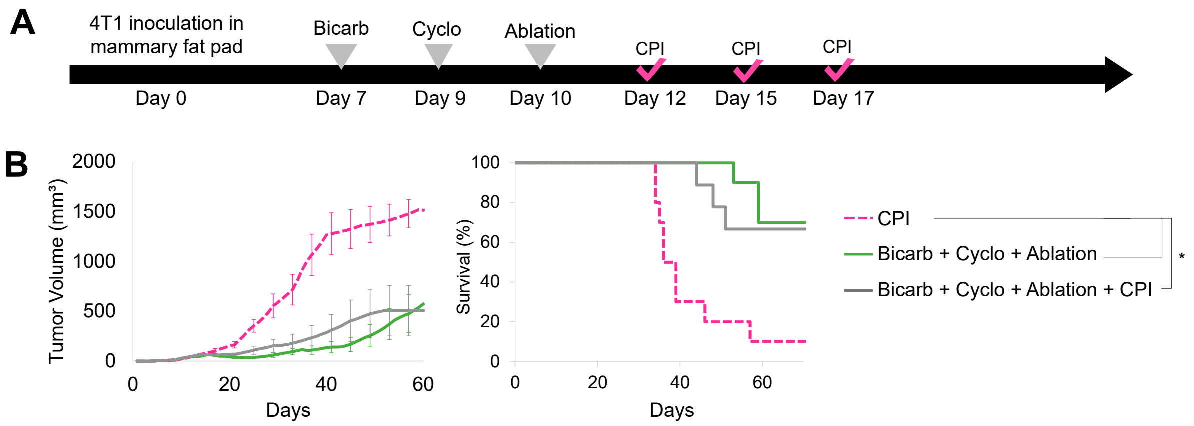

Figure S2: BiCyclA outperforms CPI in 4T1-Luc tumors. (**A**) Treatment timeline of BiCyclA (n=10), CPI (n=10), and BiCyclA + CPI (n=9). (**B**) Tumor growth and survival of mice with 4T1-Luc tumors. *P<0.05
